# Monitoring and evaluation of mental health and psychosocial support programs in humanitarian settings: a scoping review of terminology and focus

**DOI:** 10.1186/s13031-018-0146-0

**Published:** 2018-03-19

**Authors:** Jura L. Augustinavicius, M. Claire Greene, Daniel P. Lakin, Wietse A. Tol

**Affiliations:** 1 Department of Mental Health, Johns Hopkins Bloomberg School of Public Health, 624 N. Broadway, Baltimore, Maryland 21205 USA; 2 Peter C. Alderman Foundation, Mawanda Rd, Plot #855, Kampala, Uganda

**Keywords:** Monitoring and evaluation, Mental health and psychosocial support, Humanitarian settings, Low and middle-income countries, Logical framework

## Abstract

**Background:**

Monitoring and evaluation of mental health and psychosocial support (MHPSS) programs is critical to facilitating learning and providing accountability to stakeholders. As part of an inter-agency effort to develop recommendations on MHPSS monitoring and evaluation, this scoping review aimed to identify the terminology and focus of monitoring and evaluation frameworks in this field.

**Methods:**

We collected program documents (logical frameworks (logframes) and theories of change) from members of the Inter-Agency Standing Committee Reference Group on MHPSS, and systematically searched the peer-reviewed literature across five databases. We included program documents and academic articles that reported on monitoring and evaluation of MHPSS in low- and middle-income countries describing original data. Inclusion and data extraction were conducted in parallel by independent reviewers. Thematic analysis was used to identify common language in the description of practices and the focus of each monitoring and evaluation framework. Logframe outcomes were mapped to MHPSS activity categories.

**Results:**

We identified 38 program documents and 89 peer-reviewed articles, describing monitoring and evaluation of a wide range of MHPSS activities. In both program documents and peer-reviewed literature there was a lack of specificity and overlap in language used for goals and outcomes. Well-validated, reliable instruments were reported in the academic literature, but rarely used in monitoring and evaluation practices. We identified six themes in the terminology used to describe goals and outcomes. Logframe outcomes were more commonly mapped to generic program implementation activities (e.g. “capacity building”) and those related to family and community support, while outcomes from academic articles were most frequently mapped to specialized psychological treatments.

**Conclusions:**

Inconsistencies between the language used in research and practice and discrepancies in measurement have broader implications for monitoring and evaluation in MHPSS programs in humanitarian settings within low- and middle-income countries. This scoping review of the terminology commonly used to describe monitoring and evaluation practices and their focus within MHPSS programming highlights areas of importance for the development of a more standardized approach to monitoring and evaluation.

## Background

Humanitarian crises, such as disasters triggered by natural hazards and armed conflicts, are associated with high levels of psychological distress and increased risk for a range of mental disorders [1–3]. In response, mental health and psychosocial support (MHPSS) programs are increasingly being recognized as critical elements of humanitarian response. Consensus on best practices for MHPSS in humanitarian settings have been published by the Interagency Standing Committee (IASC) and the Sphere project [4, 5].

The IASC guidelines on MHPSS offer recommendations written by a task force of 27 agencies, including United Nations agencies, (international) non-governmental organizations, and the International Federation of Red Cross and Red Crescent Societies [6]. After the publication of the guidelines, an IASC Reference Group on MHPSS in Emergency Settings (hereafter referred to as the IASC reference group) was established to coordinate dissemination and implementation of the guidelines [4]. In addition to the guidelines, this reference group has produced a number of tools for MHPSS practitioners, including needs and resource assessment tools [7] and recommendations for ethical research practice [8].

The IASC MHPSS guidelines include monitoring and evaluation guidance as part of minimum response in humanitarian settings. Monitoring is defined as “the systematic process of collecting and analysing information to inform humanitarian decision making related to ongoing or potential new activities”, and evaluation is defined as “the evaluation of the relevance and effectiveness of ongoing or completed activities” [4]. By including monitoring and evaluation as a component of minimum response, the IASC MHPSS guidelines assert that monitoring and evaluation are high-priority activities that should be implemented as soon as possible in an emergency. Monitoring and evaluation activities are critical to facilitating programmatic learning and to providing accountability to stakeholders, including program participants, facilitators and funders. The IASC MHPSS guidelines provide recommendations on the general approach to monitoring and evaluation of MHPSS programs, but do not provide detailed guidance on which goals, outcomes, and indicators may be useful.

In 2014, the IASC reference group started an initiative to develop recommendations for monitoring and evaluation of MHPSS programs, with an interest in developing a common framework for use across humanitarian agencies. The initiative applied a logical framework approach. Logical frameworks (logframes) are often developed hierarchically, starting with the broad goal and then moving on to specific activities. In a logical framework approach, *goals* (or *impacts*) refer to the highest-level long-term result of having achieved an overall objective, potentially through a portfolio of projects. *Outcomes* are the overall changes that can be directly traced to a specific project and that contribute to the overall goal. *Indicators* are units of measurement used to measure the impact of a goal (i.e., impact indicators) or of an outcome (i.e., outcome indicators). *Means of verification* are tools used to quantitatively or qualitatively measure indicators [9–11]. In practice, MHPSS organizations use a variety of terms to describe the components of logical frameworks. For example, “outcomes” are often referred to as “objectives” and “indicators” can be described as “targets”.

This article describes a scoping review that was commissioned as part of the IASC reference group’s effort to develop a common framework for monitoring and evaluation of MHPSS programs. Such a common framework may be helpful for several reasons. First, systematic monitoring and evaluation may contribute to bridging the gap between research and practice in the MHPSS field. In practice, the most commonly applied MHPSS interventions are primarily non-specialized and community-based supports. In contrast, the focus of MHPSS intervention research has predominantly been on examining the effectiveness of more specialized interventions for people with symptoms of posttraumatic stress disorder, depression, and anxiety [12]. Use of a common framework for monitoring and evaluation as part of humanitarian programming may facilitate the development of practically relevant knowledge for MHPSS activities selected by humanitarian agencies, as opposed to MHPSS activities that reflect the interests of researchers.

Second, a common framework for monitoring and evaluation may assist in consolidating learning across humanitarian agencies. Currently, humanitarian agencies use a range of different terms to describe impacts and outcomes of MHPSS activities and apply different indicators to assess progress on these impacts and outcomes. The use of different terminology and indicators inhibits learning across programs with similar objectives implemented by different humanitarian agencies. Over time, the application of a common monitoring and evaluation framework by humanitarian agencies would help to identify which types of program activities were successful in reaching similar MHPSS goals.

The purpose of this scoping review was to examine monitoring and evaluation terminology and the focus of MHPSS programs in humanitarian settings in LMIC. We focused on LMIC because the large majority of populations affected by humanitarian crises reside in LMIC [13] and because monitoring and evaluation concepts in high-income countries may differ from those applied in low-resource settings. Our scoping review was guided by two research questions: (1) Which common trends may be observed in the terminology used to describe MHPSS program goals (impacts), outcomes, their indicators, and their means of verification, both in humanitarian documents and the peer-reviewed literature?; and (2) what is the focus of monitoring and evaluation within goals (impacts), outcomes, and their indicators?

## Methods

Given the aim of the scoping review (i.e., identifying common monitoring and evaluation terminology and the focus of monitoring and evaluation frameworks for MHPSS programs in humanitarian settings in LMICs, as opposed to synthesizing outcomes of evaluation studies), a range of studies and program documents were included. Scoping reviews often “map” the existing focus within an area of study in order to identify gaps in knowledge, summarize research trends, and describe the scope of an area of study [14, 15].

### Search strategy

Logframes and theory of change documents (referred to hereafter as logframes) were collected on a voluntary basis from member agencies of the reference group. In collecting program documents, this study sought to represent MHPSS activities across agencies working within various humanitarian sectors in which MHPSS programming is often embedded (i.e., health, protection, education, nutrition, and camp management and coordination). A call was put out to agencies through the IASC reference group’s email list and logframes were sent to the senior author (WT) by email.

The search for academic articles was restricted to peer reviewed articles describing monitoring and evaluation of MHPSS programs in humanitarian settings in LMIC. Searches were carried out in EMBASE, PILOTS, PsycInfo, PubMed/MEDLINE, and the WHO regional databases. Databases were searched from their dates of origin to January 11, 2015. We applied search terms under four broad categories across the databases: “low- and middle-income countries” AND “monitoring and evaluation” AND “humanitarian settings” AND “mental health and psychosocial support interventions”. The detailed search strategy for PubMed/Medline can be found in Supplemental Materials. As part of our searches we identified relevant (systematic) reviews. These were hand searched for further identification of relevant documents.

### Inclusion and exclusion

All logframes were anonymized, and duplicates were removed for both program documents and studies identified in the peer-reviewed literature. Subsequently, all identified documents were screened for relevance. Logframes and academic articles were screened in parallel by two independent reviewers. Screening of the logframes was comprised of reading of the complete document. Screening of academic articles was comprised of two phases. In the first, study inclusion was based solely on titles and abstracts, and in the second, inclusion was based on the full-texts of articles. If the two reviewers did not agree on the decision to include or exclude an article, a third reviewer was consulted.

Logframes and academic articles were included for review if: (1) they contained original data from an MHPSS program; (2) detailed monitoring and evaluation was conducted (e.g., using qualitative, quantitative, or both qualitative and quantitative approaches); and (3) they focused on study populations exposed to a humanitarian crisis in a LMIC. Academic articles not describing original data (e.g. advocating for certain monitoring and evaluation strategies), as well as general advocacy papers, letters to the editor, and book reviews were excluded, as were studies describing monitoring and evaluation programs in settings of chronic adversity in the absence of humanitarian crisis. No restriction was placed on the age of the study population. Logframes and academic articles in a language not spoken by a member of our study team were excluded. Our study team was fluent in English, French, Spanish, German, and Dutch. The operational definitions used for the purposes of inclusion and exclusion are described in Table 1.

**Table 1:** Operational definitions used for inclusion and exclusion of logframes and academic articles

Monitoring and evaluation	Monitoring can be defined as a continuing function that aims to provide the management and main stakeholders of an ongoing intervention with early indications of progress, or lack thereof, in the achievement of results. Evaluation is a selective exercise that attempts to systematically and objectively assess progress towards and the achievement of an outcome [10]
Mental health and psychosocial support (MHPSS)	Any local or outside support that aims to protect or promote psychosocial well-being and/or prevent or treat mental disorder [4]
Humanitarian settings	Areas affected by a broad range of emergencies, including natural disasters, armed conflicts including wars, and technological and industrial disasters
Low and middle-income countries (LMIC)	Classification of countries in accordance with estimations of Gross National Income

### Data extraction

Data extraction forms were piloted among reviewers before extraction began. All data were double extracted in parallel by two independent reviewers and discrepancies were resolved by involving a third reviewer. From both logframes and academic articles we extracted the following: (1) descriptions of the program’s overall goal (impact) and outcomes (or similar level objectives using different types of monitoring and evaluation language); (2) terminology used to describe indicators for impacts and outcomes; and (3) means of verification for the indicators.

In addition, we extracted information on geographic region; type of humanitarian setting; age range of MHPSS participants; MHPSS activities implemented; and monitoring and evaluation approach. In accordance with UNICEF’s categorizations, geographic regions were categorized as: Central and Eastern Europe and the Commonwealth of Independent States; East Asia and the Pacific; South Asia; Eastern and Southern Africa; Middle East and North Africa; West and Central Africa; and Latin America and the Caribbean. Types of humanitarian settings were grouped as current armed conflict; post-conflict; refugee settings; disasters triggered by natural hazards; and disasters triggered by technological hazards. The recipients of MHPSS interventions were classified as children and adolescents (below 18 years), adults (18 years and older), or a mixture of both. The intervention name or a short description was extracted to describe the MHPSS activities reported in the academic articles. Monitoring and evaluation approach was classified as quantitative, qualitative, or both quantitative and qualitative.

The primary objective of the study was not to review program results themselves, but to review the terminology used in describing programmatic approaches to monitoring and evaluation and the primary focus of monitoring and evaluation practices. A quality appraisal assessment was therefore not conducted.

### Thematic analysis

Data analysis involved both inductive (or “bottom-up”) and deductive (or “top-down”) approaches. The “bottom-up” analysis employed thematic analysis to (1) identify common trends in the language used to describe goals (impacts), outcomes, indicators, and means of verification; and (2) to examine common themes in content across goals (impacts), outcomes, and their indicators. Once grouped, the language used for goals, outcomes, indicators, and means of verification was compared across all content areas. Common themes and subsequent codes were then iteratively developed across each logframe component. First, we read all of the documents to gain an understanding of overall content. Second, we conducted an analytic reading of the data, in which we annotated descriptions of goals, outcomes, and their indicators. Third, we re-read the descriptions of goals, outcomes, and their indicators and looked for repetition within outcomes and indicators. In the third step we developed a code book. Fourth, we labeled all outcomes and indicators using codes from the code book. Analysis involved describing, comparing, and categorizing the coded data in order to conceptualize themes within outcomes and indicators.

A deductive (i.e. “top-down”) analysis process was carried out using the 4 W’s (Who is Where, When, doing What in MHPSS) tool in order to map the focus of the outcomes in our sample of monitoring and evaluation frameworks [16]. The 4 W’s tool was designed for organizational purposes to map MHPSS activities across broad geographic regions in emergency settings in an effort to improve coordination among organizations working in the sector [16]. The tool does not represent best practices. The IASC 4 W’s tool describes 11 activity codes, many of which correspond to action sheets in the IASC guidelines [4]. Activities are categorized as community-focused, person-focused, and general [16]. A double coding procedure was used in which at least two of the authors examined and mapped outcomes from logframes and academic articles to the 4 W’s activity codes. These codes were subsequently reviewed by a panel of the authors for agreement and consistency. In the event of a coding discrepancy, a third reviewer was consulted.

### Consultations

Review consultations were carried out with a subset of members of the reference group at various stages throughout the review process. The first consultation occurred in June 2014 and was focused on preliminary analysis of goals and outcomes that had been extracted during the first phase of the review. Based on this consultation, it was decided that indicators and means of verification should be extracted, and that the review should be updated in a subsequent phase. As a result, the review and analyses were updated in May 2015 and a second consultation was held in November 2015. Analyses were again updated in December 2016 in preparation for publication.

## Results

**Fig. 1:**
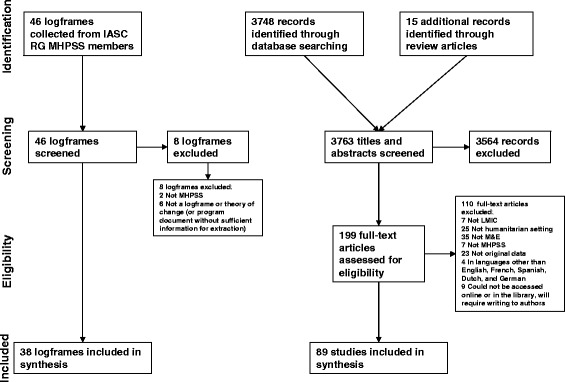
Flow of logframes and academic articles through the phases of review

The flow diagram in Fig. 1 shows the process through which 38 logframes and 89 academic articles were included in the scoping review. Of the 46 program documents submitted by humanitarian agencies, 33 logframes, 2 theories of change, and 3 other program documents were included. Table 2 describes characteristics of the MHPSS programs and their participants across both types of documents. The largest share of logframes (59%) and academic articles (43%) described programs in Africa and the Middle East. A substantial percentage of logframes described programs in refugee settings (40%), while academic articles were more commonly focused on post-conflict settings (40%). Almost half the logframes described programs targeting people of all ages (47%), however a third of logframes did not specify the age of the program’s target population (37%). Approximately one third of the academic articles described programs for adults (35%) and for children and adolescents (35%), with many programs focusing on both age groups combined (25%). A quantitative approach to monitoring and evaluation was more common both in logframes (55%) and in the academic literature (49%) as compared to a qualitative approach or an approach using both quantitative and qualitative methods.

**Table 2:** Characteristics of MHPSS programs and their participants

	Logframes (*n* = 38)	Academic Articles (*n* = 89)
Geographic Region, No. (%)		
Central and Eastern Europe and the Commonwealth of Independent States	2 (5.1)	22 (22.2)
East Asia and the Pacific	0	13 (13.1)
South Asia	4 (10.3)	11 (11.1)
Eastern and Southern Africa	13 (33.3)	15 (15.2)
Middle East and North Africa	7 (17.9)	15 (15.2)
West and Central Africa	3 (7.7)	13 (13.1)
Latin America and the Caribbean	2 (5.1)	10 (10.1)
Not reported	8 (20.5)	0
Humanitarian Setting, No. (%)		
Ongoing armed conflict	8 (21.1)	6 (6.7)
Post conflict	2 (5.3)	36 (40.4)
Refugee settings	15 (39.5)	10 (11.2)
Natural disasters	3 (7.9)	27 (30.3)
Technological disasters	0	0
Other	1 (2.6)	10 (11.2)
Not reported	9 (23.7)	0
Age, No. (%)		
Children and adolescents (< 18 years)	5 (13.2)	31 (34.8)
Adults (≥ 18 years)	1 (2.6)	31 (34.8)
Combination of children, adolescents, and adults	18 (47.4)	22 (24.7)
Not reported	14 (36.8)	5 (5.6)
Monitoring and evaluation Approach, No. (%)		
Quantitative	21 (55.3)	22 (24.7)
Qualitative	0	44 (49.4)
Both quantitative and qualitative	12 (31.6)	16 (18.0)
Not reported	5 (13.2)	7 (7.9)

The total number of regions is greater than the total numbers of logframes and articles, respectively, since multiple regions were reported in one logframe or article in some cases

### Trends in description of goals (impacts), outcomes, indicators, and means of verification

#### Lack of distinction between overall goal and outcomes and between impact and outcome indicators

The majority of academic articles described an evaluation of a specific intervention program as opposed to program monitoring. Within this context and the expected framework of an academic article, authors of academic articles frequently described the goal of their study, but not the goal of the overall program of which the specific intervention may have been a part. Moreover, the language describing program goals (the overall result to which a project may contribute) and outcomes (the specific objective(s) that the project aims to achieve) was often similar within an article, which made it difficult to differentiate between pieces of information pertaining to these two levels of the logical framework. Surprisingly, the latter finding was also common among logframes. While information was intentionally categorized by program designers at goal and outcome levels within the logframes, the actual language used was frequently comparable. For example, one program goal and a corresponding outcome were “*to effectively meet the needs of people affected by the crisis*” and “*to respond to the most urgent needs of men, women, boys, or girls affected by the crisis*”. Impact indicators were reported in a third of logframes, were identified in less than 5% of academic articles, and were similarly difficult to differentiate from outcome indicators, particularly among the academic articles. For example, impact and outcome indicators extracted from the same academic article were “*Significant improvements of well-being at the individual and population levels*” and “*Status of psychosocial well-being as measured through the psychosocial assessment instrument*”, respectively.

#### A lack of specificity

Across study documents, outcomes were frequently related to program expectations in general, but were less detailed regarding the direction of change. Among outcomes that included language pertaining to the direction of change, such as to “*promote*”, “*support*”, “*reduce*”, and “*alleviate*”, many did not describe a final desired state. For example, one outcome was “*to prevent and reduce mental health problems due to war conditions*”. While measurable quantities or qualities were included in the majority of logframe indicators, on occasion, indicators were described without reference to measurable quantities or qualities. For example, one logframe indicator was “*Interaction and empathic connection to others*”. The majority of indicators across documents did not make any reference to a time frame during which the specified change would occur.

#### Contrasting means of verification

Both logframes and academic articles described quantitative and qualitative means of verification. The means of verification for quantitative indicators described in program documents differed from those used in academic articles. In reference to quantitative indicators, logframes frequently reported generic, non-standardized means of verification such as photos, updates, reports, assessments, work plans, questionnaires, or health facility records. Some logframes did report specific, standard means of verification for quantitative indicators, such as a “7-item resilience scale” and the Camberwell Assessment of Needs Appraisal Schedule [17]. Academic articles described a plethora of scales used to measure a wide range of quantitative indicators, however psychometrically tested scales were rarely explicitly described in the logframes. Examples of the means of verification described in academic articles include the Beck Depression Inventory [18] and the Child Behavior Checklist [19]. Qualitative means of verification, such as interviews and focus group discussions, were used in both logframes and academic articles although formal process evaluation methods were rarely described.

### “Bottom-up” analysis: The focus of goals, outcomes, and their indicators

Through thematic analysis, we identified six distinct themes in terminology used to describe goals, outcomes, and their indicators across logframes and academic articles. The first theme encapsulated programs seeking to promote individual resilience and psychosocial well-being, and prevent mental health and psychosocial problems. This theme emerged more often among logframes than among academic articles. Programs with goals and outcomes focusing on the first theme intended to support or enhance individual-level resilience most commonly by providing community-level supports. Examples of the language used for goals, impact indicators, outcomes, and outcome indicators for this theme included “*to enhance community structures*”, “*improved empowerment*”, “*to increase hope*”, and “*the number of prevention programs implemented*”, respectively.

The second theme described programs seeking to reduce particular mental health and psychosocial symptoms and functional impairment. This was a more dominant theme among academic articles. Examples of the language used to describe goals, impact indicators, outcomes, and outcome indicators for the second theme were “*to improve mental health symptoms and functioning through care*”, “*decreased prevalence of MHPSS problems*”, “*to reduce symptoms and distress*”, and “*percent reduction in symptoms*”, respectively.

The third theme applied to programs that primarily aimed to build capacity to identify, intervene on, and monitor mental health and psychosocial problems. This theme often applied to both logframes (e.g. through integration of services) and to academic articles (e.g. through training). Examples of terminology used for goals and impact indicators included “*to build capacity for mental health services*” and “*MHPSS knowledge dissemination between stakeholders*”. Examples of the language used for outcomes and outcome indicators included “*to promote sustainability through training of trainers*” and “*number of trained MHPSS providers*”, respectively.

The fourth theme described programs that focused centrally on enhancing environments in which child development can flourish. Although related, this theme was differentiated from the second theme as it applied to programs that specifically attempted to support or enhance community-level systems (e.g. community resilience) rather than individual resilience, and focused on children and adolescents. The fourth theme included definitions of child development within social, economic, and physical health domains, and more commonly emerged from logframes. Examples of the terminology used for goals for the fourth theme included “*to provide an environment where individuals are able to grow and thrive*” and “*for people with mental health problems to be included in the community*”. The language used for impact indicators included “*involvement of parents, teachers, and communities in children’s education” and “community awareness of MHPSS problems*”. Examples of terminology used for outcomes and outcome indicators included “*to promote child development through parental support*” and “*children’s cognitive improvement*”, respectively.

The fifth theme applied to programs describing macro-level goals and outcomes, for example to build peace between groups in post-conflict settings, and to address structural problems within societies. This theme emerged less frequently from the data as compared to the other themes and was primarily described in logframes. Examples of the language used in goals for the fifth theme included “*to address underlying structural inequalities*” and “*to enable a participatory and human rights respecting democratic restoration of the social fabric*”. “*Change in policy*” was an example of the terminology used for an impact indicator. Outcome-level language included “*to support peaceful co-existence between and within communities*” and “*to foster collective trauma healing as part of peacebuilding*”. An example of the language used for an outcome indicator was “*improvement in social fabric*”.

The sixth theme described programs that sought to protect vulnerable groups of people, such as women, children, the elderly, and people with disabilities. This theme almost exclusively emerged from logframe data. The terminology used for goals and impact indicators for the sixth theme included “*to protect vulnerable groups*” and “*number of families successfully re-unified*”. Examples of the language used in outcomes and outcome indicators included “*to reduce the risk of interpersonal violence including sexual and gender-based violence*” and “*percent of sexual and gender-based violence survivors who access safe spaces*”.

### “Top-down” analysis: MHPSS activities within outcomes

**Fig. 2:**
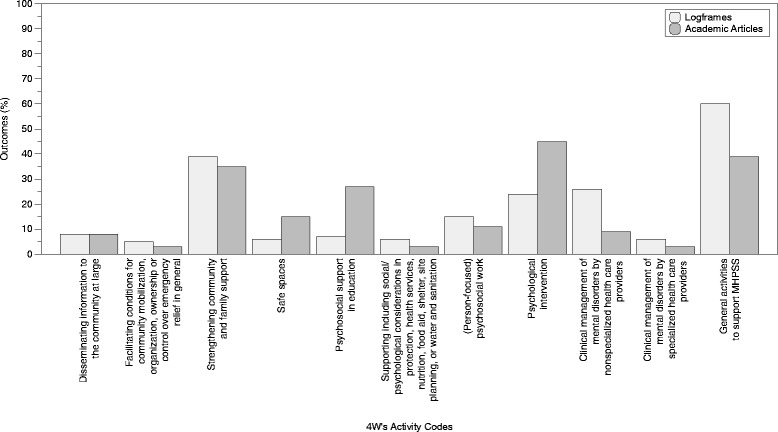
Proportion of outcomes mapped to the 4Ws activity. Light gray bars represent the proportion of logframe outcomes and dark gray bars represent the proportion of outcomes from academic articles

In addition to the inductive thematic analysis, we were interested in mapping the focus of outcomes in logframes and academic articles to common MHPSS activities. We identified 85 distinct outcomes in the logframes and 159 outcomes in academic articles. These were mapped onto the activities of the IASC 4Ws tool. The proportions of outcomes mapped to each of the 11 4 W’s activity codes [16] are shown in Fig. 2. Of the 85 outcomes described in logframes, the most commonly applied activity codes were “*general activities to support MHPSS*” (60%) and “*strengthening community and family supports*” (39%). The largest proportion of the 159 outcomes described in academic articles were mapped to the “*psychological intervention*” activity code (45%).

## Discussion

Monitoring and evaluation is critical to the evolution of MHPSS in humanitarian settings. The development of indicators for monitoring and evaluation in MHPSS was ranked as the top fourth most important research priority in a recent consensus-based setting of a research agenda [20]. The current scoping review was performed as part of an initiative to develop a common framework for monitoring and evaluation of MHPSS in humanitarian settings by a group of international humanitarian agencies. The aims of this scoping review were to support this initiative by identifying common trends in the terminology used to describe goals, outcomes, indicators and means of verification, and by examining the focus of goals, outcomes, and indicators.

In combining both programmatic and research documents, and by including academic articles from a range of databases, we believe this scoping review provides broader insight into the terminology used in monitoring and evaluation frameworks and the focus of these frameworks within the MHPSS field. Despite these strengths, this review has several limitations. Program documents were drawn from within the IASC reference group and only represent a small proportion of all MHPSS program documents that exist, introducing selection bias as those agencies more confident in their programming may have been more likely to respond, may have only submitted logframes of the highest quality, and may have submitted more than one logframe. Our review of published academic literature may also have resulted in selection bias since published studies may have been more likely to use a more restricted range of means of verification or those quantitative means of verification with particular psychometric properties compared to studies that were not published in peer reviewed journals. In addition, the search of the academic literature used English keywords and only articles written in five languages were included for review. Database searches were also limited to the fields of medicine, psychology, social work, public health, and nursing. All data was extracted to fit within a logical framework format, however our results should be interpreted with an understanding that academic articles are not typically written to fit such a format. No comprehensive search of the grey literature was performed as part of this scoping review, representing an important limitation. In order to gain rapid insight into monitoring and evaluation in practice, organizations were asked directly for logframes and these logframes represent only a small portion of the grey literature.

In the remainder of this section we discuss the key findings of the scoping review and their implications for MHPSS monitoring and evaluation practice. First, we noted a lack of specificity in how goals and outcomes were phrased. A strong overlap in goals and outcomes, or a lack of differentiation between these two levels, may suggest challenges in conceptualizing a common overall goal for MHPSS practitioners. According to logical frameworks, the goal is intended to be a high-level achievement that is not necessarily attained by one program, but by a portfolio of programs. Each outcome is defined as an achievement set for a specific program. Within the field of project management, the inconsistent use of terminology in logframes has been one of the chief criticisms of this approach [21–23]. In the MHPSS field this challenge may be related to the fact that improvements in mental health and well-being are often conceptualized as the key focus of MHPSS activities (hence included as an outcome), yet because mental health has many influences, mental health and well-being may also be perceived as higher order constructs that require a portfolio of program activities to achieve (hence they can also be included as a goal). For example, water and sanitation, protection, education, as well as health programs may all be conceptualized to contribute to overall well-being. Within monitoring and evaluation more broadly, however, attention has been drawn to the use of unclear terminology at the goal and outcome level [21] indicating that this issue is not unique to MHPSS. A lack of differentiation between terms may also point to a lack of training. Training in monitoring and evaluation is needed so that those implementing programs understand how logframes should be developed, specifically how terminology for the individual components of logframes (i.e. goals, outcomes, indicators) should be used and how both quantitative and qualitative means of verification should be selected and administered.

Second, we noted a lack of specificity in language used to describe outcomes and indicators. The IASC guidelines suggest that indicators should be SMART (Specific, Measurable, Achievable, Relevant, and Time-bound) and that for feasibility, only a few indicators should be selected for each program [4]. In contrast, we found that indicators were commonly stated using similar language. In addition to a lack of specificity in the language used for many indicators, we also found that indicators rarely made reference to time. This may reflect a lack of consensus or confusion regarding the definition and format of these components of the logical framework which would confirm the importance of developing more specific monitoring and evaluation guidance for use by humanitarian agencies. Considering these challenges, a common monitoring and evaluation framework should include clear operational definitions at each logframe level that are flexible enough to be adapted for different kinds of programs. Providing examples of appropriately worded goals, outcomes, and indicators may also help clarify and unify frameworks across organizations. Specific recommendations and examples are provided in the newly released field test version of the common monitoring and evaluation framework for MHPSS in emergency settings released by the IASC Reference Group on MHPSS [11].

Third, we draw attention to the finding that psychometrically tested instruments were frequently described in the peer-reviewed literature as means of verification, but that these were not commonly applied in the program documents. Among programs assessing quantitative indicators, problems that may stem from using non-validated instruments for quantitative indicators in monitoring and evaluation have been previously noted [24], pointing to a gap between science and practice. This may reflect the different resources available to teams implementing MHPSS monitoring and evaluation under time and resource constraints as opposed to those available to teams with dedicated resources for research [25]. The relatively low use of validated means of verification to track quantitative indicators in practice despite their widespread availability and feasibility in programming indicates the potential utility of a user-friendly overview of psychometrically sound tools that have been validated in various contexts. This finding also calls for researchers and practitioners to work together to identify and adapt tools that more closely meet real-world needs. In parallel, the relative importance of qualitative process evaluation should not be overlooked in research nor practice. Process evaluations should supplement outcome evaluations in order to contribute to the evidence base and inform policy and practice by examining program implementation, mechanisms of impact, and contextual factors that influence program delivery and operations [26]. Both quantitative evaluation of programs using validated instruments and qualitative process evaluations are integral to the success of monitoring and evaluation frameworks.

Fourth, we note that our “bottom-up” analysis yielded broadly similar themes across content of both program documents and academic articles. While very few academic articles described monitoring and evaluation of programs pertaining to the theme of (child) protection and human rights, there were many program documents to which this theme applied. Several logframes described programs aimed toward peace-building, which was less commonly described in academic articles. As previously discussed as a limitation, this observation may reflect the content of the five databases we selected for our searches. At the same time, it may also reflect less emphasis in these fields on publishing monitoring and evaluation efforts in peer-reviewed journals, and for instance, a stronger focus on activism and implementation, or an emphasis on types of monitoring and evaluation techniques that may not be easy to publish in peer-reviewed journals (e.g. favoring qualitative, experiential methods that are not intended to be generalizable). Regardless of the cause of this discrepancy, consensus on the importance of MHPSS activities across a broad range of sectors, including (child) protection, suggests that it will be important for efforts in developing a common monitoring and evaluation framework to understand and allow for different program activities and methodological preferences.

Fifth, we carried out a mapping exercise where outcomes from logframes and peer-reviewed articles were mapped to the IASC’s 4Ws activity codes [16, 27] in a non-mutually exclusive manner. Logframe outcomes were most commonly mapped to “*general activities to support MHPSS*” and “*strengthening community and family supports*”. This finding is not surprising given that indicators listed in the Sphere Handbook section on mental health service provision focus on activities such as training, staffing, and medication availability [5]. In contrast, academic articles were most commonly mapped to the “*psychological intervention*” activity code. A recent review of the academic literature on public health interventions in humanitarian settings found that the majority of MHPSS publications focused on psychological interventions and that studies on psychosocial interventions offered weaker evidence and were of lower quality [25]. These findings are in keeping with previous research showing that MHPSS programming tends to focus on non-specialized services and community-based supports, whereas academic research is primarily focused on assessing the effectiveness of more specialized services and intervention protocols [12, 28]. Taken together, these findings echo the call for increased engagement of researchers with agencies working in the humanitarian sector as well as improved knowledge and data sharing across research and practice [25].

## Conclusions

The purpose of this scoping review was to explore the language used to describe MHPSS program level goals, impact indicators, outcomes, outcome indicators, and means of verification as reported in logframes, theories of change, and published articles. We also sought to examine the focus of program goals, outcomes and indicators, and to investigate how outcomes from this sample of logframes and academic articles could be mapped to general categories of MHPSS activities. We identified conceptual gaps between research and practice ranging from ambiguous language used when describing program goals and outcomes to heterogeneity in measurement and instrument quality, and found six common themes across goals, outcomes, and indicators. The results of the “top-down” analysis highlight continued differences in the areas of focus in practice and in research. These conclusions may help inform the development of monitoring and evaluation frameworks for MHPSS in humanitarian settings.

## Abbreviations

4Ws: Who is Where, When, doing What in MHPSS tool
IASC: Inter-Agency Standing Committee
LMIC: Low and Middle-Income Country
Logframe: Logical Framework
MHPSS: Mental Health and Psychosocial Support
